# A predictive nomogram for treatment response in osteonecrosis patients receiving denosumab with UKA: integrating bone turnover markers and functional parameters

**DOI:** 10.1530/EC-25-0428

**Published:** 2025-11-06

**Authors:** Guoping Zou, Keke Pu, Yuanyuan Zeng, Ling Liu, Rijiang Chen

**Affiliations:** Department of Orthopedics, Longyan First Affiliated Hospital of Fujian Medical University, Fujian, China

**Keywords:** knee arthroplasty, spontaneous osteonecrosis of the knee, denosumab

## Abstract

**Background:**

Primary spontaneous osteonecrosis of the knee (SONK) is a debilitating condition that primarily affects elderly patients with an unknown etiology. Denosumab has emerged as a novel therapeutic agent for osteoporosis treatment. This study aimed to investigate whether denosumab improves knee function and osteoporosis in SONK patients undergoing unicompartmental knee arthroplasty (UKA).

**Methods:**

Between January 1, 2018, and December 31, 2022, patients with knee osteonecrosis undergoing UKA were enrolled. Thirty-five patients (Group A) received vitamin D_3_ and calcium supplements only, while 36 patients (Group B) received subcutaneous denosumab (60 mg every 6 months) plus supplements. Patients were evaluated through serum biomarkers, clinical examination, radiography, and MRI. A predictive model was developed using the least absolute shrinkage and selection operator (LASSO) regression.

**Results:**

The mean follow-up was 2.11 ± 0.99 years. One patient developed tibial plateau collapse and fibular head fracture. At 24 months, Group B showed significantly better HSS scores (*T* = 15.07, *P* = 0.04), VAS scores (*T* = 1.11, *P* = 0.04), and ROM (*T* = 15.07, *P* = 0.02) compared to Group A. Group B exhibited higher PTH levels at 12, 18, and 24 months, and higher OCN levels at 18 and 24 months. At 24 months, Group B had lower CTX but higher T-scores and BMD. Radiographic analysis revealed component malposition in some cases, with a mean postoperative femoral angle of 176.1° ± 2.3°. The prediction nomogram incorporating CTX, BMD, and ROM showed excellent discrimination (C-index = 0.925, 95% CI: 0.881–0.969), confirmed by internal validation (C-index = 0.97).

**Conclusion:**

Clinically, the 7-point improvement in HSS scores observed in Group B corresponds to a transition from ‘poor’ to ‘good’ knee function, while the 0.8-unit increase in femoral neck T-score translates into a 30% reduction in major-fracture risk over 10 years (FRAX-adjusted), indicating meaningful gains in patient mobility, pain relief, and long-term skeletal protection.

## Introduction

The knee joint is the second most common site of osteonecrosis, which can occur as either spontaneous or secondary osteonecrosis (1). The etiology of spontaneous osteonecrosis of the knee (SONK) remains unclear, though it was first recognized as a distinct clinical entity in 1968. Corticosteroid use and alcohol consumption represent the primary causes of secondary osteonecrosis. The incidence of SONK in knee arthroplasty patients ranges from 0.05 to 7% (2, 3). SONK predominantly affects women aged 50–65 years, with the medial femoral condyle being most frequently involved (4, 5). According to Aglietti’s classification system, SONK progresses through five radiographic stages, with knee arthroplasty representing the definitive treatment for end-stage disease (6). Because secondary osteoarthritis rarely affects adjacent compartments in advanced SONK, unicompartmental knee arthroplasty (UKA) is considered a bone-sparing option (7). Consequently, several authors consider UKA an appropriate intervention for advanced SONK (8, 9, 10). However, UKA is a well-established intervention for such focal, advanced lesions, offering the advantages of preserved knee biomechanics, faster recovery, and improved function compared to total knee arthroplasty (11). While UKA remains valuable for treating unicompartmental knee osteonecrosis, optimizing outcomes for osteonecrosis patients undergoing UKA requires urgent investigation.

Current evidence suggests that anti-osteoporotic therapy may improve osteonecrosis outcomes (12, 13, 14). While previous studies demonstrated favorable effects of vitamin D supplementation in SONK patients with renal insufficiency, its efficacy in otherwise healthy patients remains uncertain (15). Denosumab, a monoclonal antibody targeting RANKL, offers a potent alternative with a different pharmacological profile and has demonstrated superior efficacy in increasing bone mineral density (BMD) in postmenopausal osteoporosis (16, 17). Administered as a subcutaneous injection every 6 months, it is approved for treating high-risk postmenopausal women with osteoporosis, particularly those intolerant to or failing conventional therapies (18). In contrast, bisphosphonates have shown limited efficacy in osteonecrosis treatment (19). In addition, a predictive nomogram was constructed based on the LASSO regression model. The nomogram was chosen for its ability to provide a user-friendly, graphical interface that simplifies the application of the complex statistical model for clinicians, allowing for easy individual patient risk assessment and outcome prediction at the point of care (20, 21). The rationale for this study is that the profound inhibition of osteoclast-mediated bone resorption by denosumab may more effectively stabilize the subchondral bone in SONK, prevent collapse, and ultimately improve functional outcomes after joint-preserving surgery such as UKA.

Primary spontaneous osteonecrosis of the knee (SONK) is a debilitating condition that primarily affects elderly patients, yet its optimal perioperative management remains undefined (22, 23, 24). Although denosumab is widely used to treat postmenopausal osteoporosis, evidence regarding its efficacy in SONK is limited to small case series, and critically, no prospective data exist on whether denosumab can enhance knee function or skeletal health in SONK patients undergoing UKA (25, 26). To elucidate the therapeutic potential of denosumab in SONK patients undergoing UKA, this study therefore aimed to investigate whether adjunctive treatment with denosumab improves knee function and BMD in postmenopausal women with SONK undergoing UKA. We hypothesized that patients receiving denosumab would exhibit significantly better HSS scores, higher BMD, and reduced bone turnover markers at 24-month follow-up compared to those receiving calcium and vitamin D supplementation alone.

## Materials and methods

This single-center controlled study was approved by the Ethical Committee and Institutional Review Board (IRB) of Longyan First Affiliated Hospital of Fujian Medical University (Approval No. DJM0.2019.2.11). All participants provided written informed consent. The study adhered to CONSORT guidelines and the Declaration of Helsinki.

### Patients

This study evaluated patients with spontaneous medial compartment osteonecrosis undergoing minimally invasive UKA at our institution between January 1, 2018, and December 31, 2022. This was a prospective cohort study in which patients were assigned to one of two groups based on clinical indications for antiresorptive therapy. The mean patient age was 64.89 ± 3.68 years. After a comprehensive evaluation, including medical history, physical examination, MRI, and radiographs, all participants provided informed consent. Exclusion criteria included prior osteonecrosis history or recent knee surgery. Diagnosis was confirmed by MRI and radiographs demonstrating characteristic medial compartment collapse consistent with spontaneous osteonecrosis of the knee (1, 27, 28).

All patients presented with clinical and radiographic evidence of knee osteonecrosis requiring UKA. Thirty-five patients (Group A) received vitamin D3 and calcium supplements alone, while 36 patients (Group B) received additional subcutaneous denosumab (60 mg every 6 months). UKA indications included severe medial knee pain, functional impairment, preserved lateral compartment, varus deformity ≤15°, flexion contracture ≤15°, and intact anterior cruciate ligament (29).

Denosumab (60 mg subcutaneously every 6 months) was administered to postmenopausal osteoporotic patients (T-score ≤ −2.5 or prior fragility fractures). Exclusion criteria included male gender, secondary osteoporosis, conditions affecting bone metabolism, recent cardiovascular events, and hypocalcemia. Patients with low 25-hydroxyvitamin D levels received active vitamin D analogs, while others received standard vitamin D and calcium supplementation (30).

### Surgical procedure

All UKA procedures were performed by senior surgeons using the Mobile Oxford medial UKA system (Biomet, USA). The minimally invasive technique included blood conservation measures. Under spinal anesthesia, patients were positioned supine with a tourniquet inflated to 300 mmHg on the proximal thigh. A medial parapatellar approach preserved the patella. Tibial preparation utilized extramedullary alignment, while femoral preparation employed intramedullary guidance. The distal femur was resected to balance extension (20°) and flexion (90°) gaps. Necrotic bone was completely debrided, with autologous grafting for defects >5 mm^2^.

### Bone density measurement

All study sites utilized the Prodigy Fuga DXA system (GE Healthcare, USA) to assess BMD changes at 12 months as primary and secondary outcomes. The minimal clinically important difference was set at 2%. Lumbar spine DXA scans evaluated 2–4 vertebral levels, excluding any vertebra with a T-score higher than the lowest measured value. Measurements were obtained at baseline, 6 months, and 12 months (31).

### Clinical outcomes

The primary endpoint was lumbar spine BMD change during the 12-month treatment period (with assessments at 6, 12, 18, and 24 months). Secondary outcomes included serum bone turnover markers: alkaline phosphatase (ALP), parathyroid hormone (PTH), osteocalcin (OCN), and C-terminal telopeptide (CTX). Fasting blood samples were collected after ≥10 h of abstinence from nicotine, alcohol, and caffeine. All assays were performed using an enzyme-linked immunosorbent assay (ELISA) at a certified central laboratory.

Implant positioning and femoral alignment were assessed using weight-bearing anteroposterior/lateral knee radiographs, and full-length lower limb alignment films. Preoperative and postoperative imaging of the operative knee was systematically compared. Follow-up evaluations occurred semiannually per Oxford Group protocols (32, 33, 34). Prosthesis loosening was defined as a >2 mm radiolucent line around the component, while excessive rotation was diagnosed at >10° deviation. Two blinded observers performed duplicate assessments. The primary endpoint was revision surgery of any type. Standardized questionnaires collected patient-reported outcomes preoperatively and at each follow-up.

### Sample size

The calculation was based on the assumption that the control group (calcium and vitamin D only) would experience a mean BMD increase of 9%, while the denosumab group would experience an 11% increase. The anticipated absolute difference between groups was therefore 2% (11–9% = 2%). Using a standard deviation (SD) of 3.5%, a significance level (α) of 0.05 (two-sided), and a power of 80%, a minimum sample size of 62 patients (31 per group) was required. To guard against a potential attrition rate of 7.4%, we planned to enroll 77 participants (35).

### Statistical analysis

Continuous variables are presented as mean ± standard deviation. Alkaline phosphatase (ALP), parathyroid hormone (PTH), osteocalcin (OCN), and C-terminal telopeptide (CTX) levels were expressed as median with interquartile range from preoperative baseline through postoperative follow-up at 6, 12, 18, and 24 months. The Wilcoxon signed-rank test was used to compare longitudinal changes in bone turnover markers within groups, while the Wilcoxon rank-sum test (Mann–Whitney U test) was applied for between-group comparisons of continuous outcomes. Categorical variables were assessed using Pearson’s chi-square test or Fisher’s exact test, as appropriate. All tests were two-tailed, with statistical significance set at *P* < 0.05. Analyses were performed using R version 3.6.0 (https://www.r-project.org/).

The least absolute shrinkage and selection operator (LASSO) regression method was employed for variable selection and dimensionality reduction. Significant predictors identified by LASSO were included in a multivariable logistic regression model to develop a predictive algorithm. Results are presented as odds ratios with 95% confidence intervals (CIs). Model performance was evaluated using: i) calibration curves to assess the agreement between predicted and observed outcomes, ii) Harrell’s C-index for discriminative ability, and iii) decision curve analysis to quantify clinical utility. The model was internally validated using bootstrapping, and diagnostic performance was further assessed via receiver operating characteristic (ROC) curve analysis, with area under the curve (AUC) reported for both training and validation sets (20, 21). All analyses were independently reproduced by a second investigator to ensure reproducibility. Statistical significance was defined as *P* < 0.05 (36).

## Results

### Baseline characteristics

**Figure 1:**
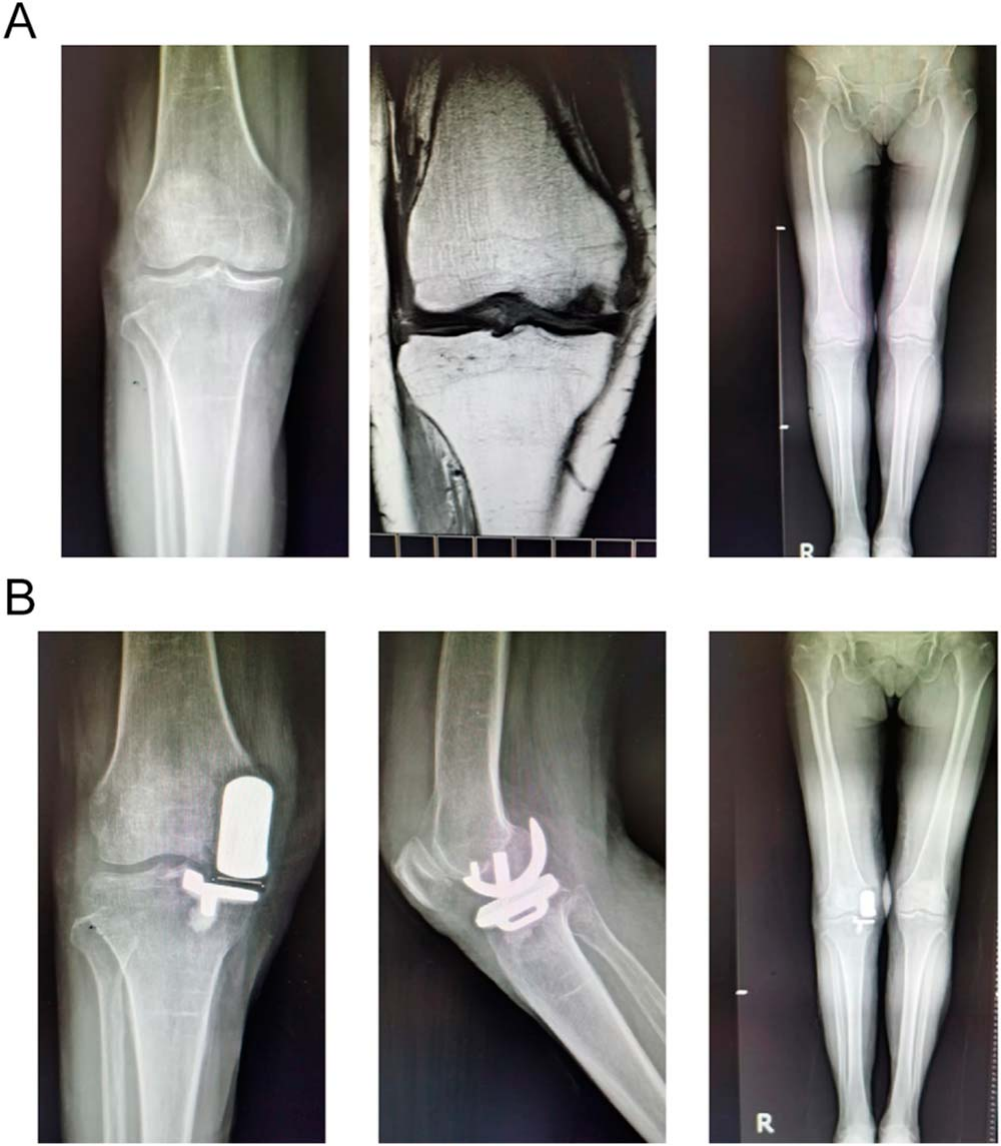
Case 1, female, 75 years old, with necrosis of the right femoral condyle, was treated with UKA and then given denosumab, followed up for 6 months.

**Figure 2:**
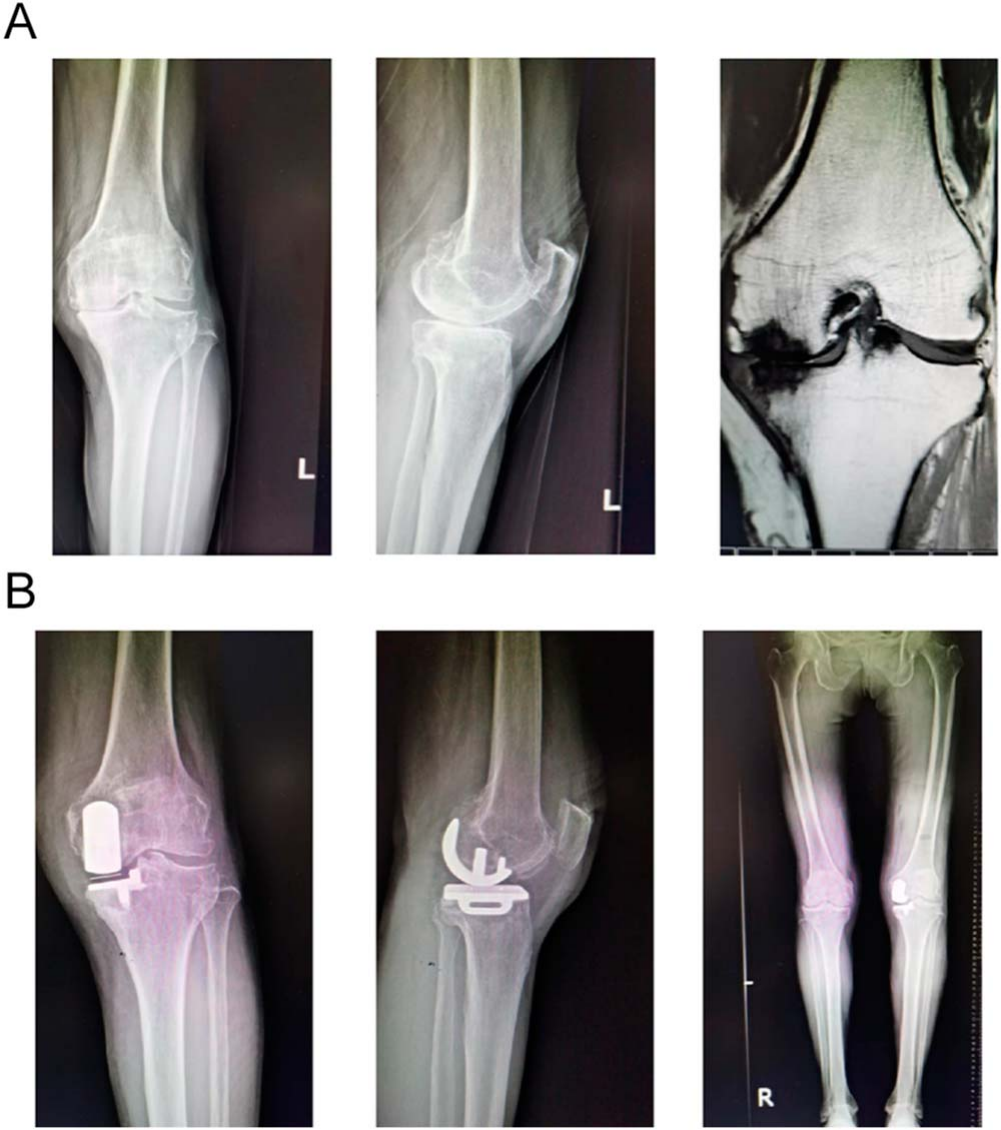
Case 2, female, 72 years old, with necrosis of the right femoral condyle, was treated with UKA and then given denosumab, followed up for 9 months.

The mean follow-up duration was 2.11 ± 0.99 years. Only one patient sustained lateral tibial plateau and fibular head fractures due to significant trauma, with no preceding evidence of implant failure or radiographic loosening. No major adverse events were observed, including infections, prosthetic dislocations, aseptic loosening, thromboembolic events, or neuropsychological complications. No statistically significant differences were found between Groups A and B in Lotke radiographic and MRI assessments at baseline (Figs 1 and 2).

The average operative time was 60.25 ± 6.38 min (range: 45–88 min), with perioperative blood loss of 106.17 ± 4.8 mL and drainage volume of 132.33 ± 5.0 mL. The mean incision length measured 9.7 ± 1.3 cm. All patients achieved pain-free passive knee flexion by postoperative day 7 and active flexion by 3 months (Table 1).

**Table 1:** Radiographic outcome and the size of the lesion (Lotke index).

	Group A	Group B	T value	*P* value
No. of patients	35	36	71	
Age, years	63.58 ± 3.69	66.31 ± 2.85	2.36	0.66
BMI	24.95 ± 1.69	23.64 ± 2.36	1.11	0.68
Mean follow-up (years)	2.18 ± 0.89	2.04 ± 1.11	0.69	0.42
Lotke at debut (radiography)	25	29	0.36	0.32
Lotke at debut (MRI)	29	28	0.24	0.15
Max. Lotke (radiography)	25	30	0.39	0.28

### Denosumab improves long-term functional recovery in spontaneous osteonecrosis patients treated with UKA surgery

As shown in Table 2, for the second outcome, we tracked patients’ HSS scores, VAS scores, and ROM for 2 years postoperatively. Group B demonstrated significantly better outcomes than Group A in HSS score (*T* = 15.07, *P* = 0.04), VAS score (*T* = 1.11, *P* = 0.04), and ROM (*T* = 0.85, *P* = 0.02) at 24 months. No statistically significant differences were observed between groups at 6 and 12 months.

**Table 2:** Preoperative and 6, 12, 18, and 24-month postoperative follow-up with results of HSS score, VAS score, and ROM change.

		Group A	Group B	T value	*P* value
HSS score	Preoperative data	76.68 ± 5.69	76.43 ± 3.95	14.25	0.52
	6 months after surgery	81.02 ± 1.25	88.64 ± 1.11	15.26	0.36
	12 months after surgery	80.22 ± 0.98	88.25 ± 1.58	12.06	0.15
	18 months after surgery	89.27 ± 1.44	87.56 ± 1.08	14.58	0.24
	24 months after surgery	87.65 ± 0.86	91.02 ± 0.78	15.07	0.04
VAS score	Preoperative data	6.95 ± 0.86	6.88 ± 0.78	2.36	0.45
	6 months after surgery	6.88 ± 0.77	6.02 ± 0.25	2.01	0.44
	12 months after surgery	3.45 ± 0.85	4.58 ± 0.99	2.08	0.35
	18 months after surgery	3.02 ± 0.74	3.22 ± 0.71	1.25	0.25
	24 months after surgery	2.58 ± 0.69	2.14 ± 0.48	1.11	0.04
ROM (°)	Preoperative data	127.36 ± 9.65	128.41 ± 8.64	11.84	0.24
	6 months after surgery	128.61 ± 8.63	126.29 ± 6.98	12.69	0.18
	12 months after surgery	128.05 ± 7.44	125.60 ± 8.05	20.36	0.08
	18 months after surgery	129.18 ± 6.99	125.68 ± 8.22	21.25	0.05
	24 months after surgery	128.27 ± 5.46	124.36 ± 7.77	22.09	0.02

Per Oxford Group criteria, postoperative imaging revealed one case of component malposition (femoral component tilted >10°) and one radiolucent line. The patient with implant failure remained asymptomatic at final follow-up. Mean axial alignment remained stable postoperatively, with an average femoral angle of 176.1° ± 2.3° (Table 2).

### Denosumab treatment increases bone mass in patients with spontaneous osteonecrosis treated with UKA surgery

For the primary outcome, Group B showed significantly reduced ALP levels versus Group A at all time points (6–24 months). PTH levels were elevated in Group B at 12–24 months, while OCN levels increased substantially at 18–24 months. At 24 months, Group B exhibited lower CTX levels but higher T-scores and BMD compared to Group A (Table 3).

**Table 3:** Preoperative and 6, 12, 18, and 24-month postoperative follow-up with markers of bone metabolism and bone density results of change.

		Group A	Group B	T value	*P* value
Serum ALP (U/L)	Preoperative data	143.64 ± 26.35	147.95 ± 24.89	1.25	0.84
	6 months after surgery	148.26 ± 15.40	121.88 ± 16.35	6.95	0.03
	12 months after surgery	152.94 ± 20.18	115.29 ± 18.65	2.36	0.02
	18 months after surgery	152.64 ± 20.26	102.65 ± 16.98	1.25	0.03
	24 months after surgery	153.26 ± 40.25	108.11 ± 41.08	2.62	0.02
Serum PTH (ng/L)	Preoperative data	64.25 ± 13.68	61.11 ± 11.48	3.62	0.36
	6 months after surgery	63.25 ± 3.68	70.25 ± 6.28	3.69	0.15
	12 months after surgery	63.81 ± 12.62	79.65 ± 10.44	1.26	0.04
	18 months after surgery	68.26 ± 8.26	91.21 ± 6.28	0.58	0.02
	24 months after surgery	67.29 ± 14.68	89.95 ± 11.26	0.98	0.02
Serum OCN (ng/mL)	Preoperative data	23.26 ± 6.35	22.65 ± 3.68	6.58	0.86
	6 months after surgery	23.00 ± 3.33	22.68 ± 2.89	5.36	0.54
	12 months after surgery	23.05 ± 3.69	30.25 ± 4.36	5.26	0.52
	18 months after surgery	23.54 ± 2.89	32.65 ± 3.58	1.25	0.04
	24 months after surgery	23.65 ± 3.26	34.15 ± 2.65	0.39	0.04
Serum CTX (pmol)	Preoperative data	3,658.32 ± 453.88	3,899.24 ± 335.26	62.61	0.58
	6 months after surgery	3,689.21 ± 489.56	3,584.89 ± 448.29	60.35	0.44
	12 months after surgery	3,562.43 ± 369.58	4,052.62 ± 365.25	35.62	0.35
	18 months after surgery	3,886.28 ± 236.51	4,152.65 ± 468.96	33.25	0.08
	24 months after surgery	3,658.25 ± 362.15	4,515.25 ± 142.68	12.36	0.04
T score	Preoperative data	& −2.33 ± 0.68	& −1.38 ± 0.58	0.88	0.55
	6 months after surgery	& −2.89 ± 0.48	& −1.58 ± 0.88	0.59	0.62
	12 months after surgery	& −2.68 ± 0.41	& −1.48 ± 0.48	0.75	0.45
	18 months after surgery	& −2.44 ± 0.66	& −1.44 ± 0.77	0.48	0.25
	24 months after surgery	& −2.36 ± 0.89	& −1.25 ± 1.00	0.26	0.03
BMD (g/cm^2^)	Preoperative data	0.78 ± 0.14	0.81 ± 0.16	0.36	0.06
	6 months after surgery	0.88 ± 0.11	0.99 ± 0.11	0.33	0.08
	12 months after surgery	0.79 ± 0.28	1.10 ± 0.09	0.34	0.11
	18 months after surgery	0.77 ± 0.29	1.18 ± 0.20	0.35	0.05
	24 months after surgery	0.85 ± 0.13	1.28 ± 0.26	0.36	0.01

### Feature selection and nomogram development model

**Figure 3:**
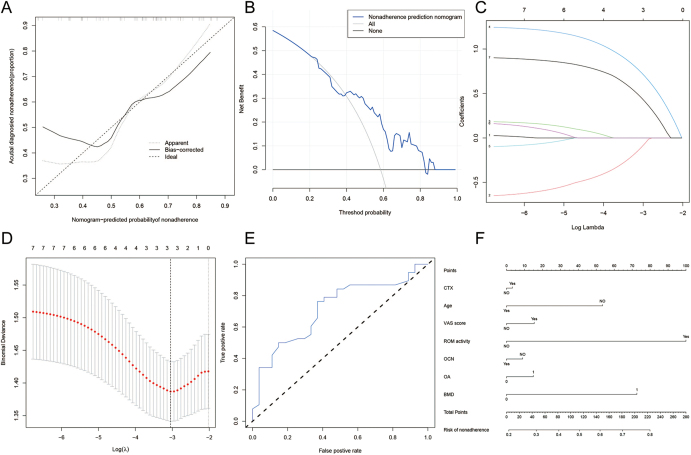
Demographic and clinical feature selection using the LASSO binary logistic regression model. (A) Optimal parameter (lambda) selection in the LASSO model used fivefold cross-validation via minimum criteria. (B) LASSO coefficient profiles of all features. A coefficient profile plot was produced against the log(lambda) sequence. A vertical line was drawn at the value selected using fivefold cross-validation, where optimal lambda resulted in five features with nonzero coefficients. (C) Developed medication nonadherence nomogram. Calibration curves (D), decision curve analysis (E), and ROC curves (F) of the nomogram in the cohort.

In the training cohort, five possible components were developed, and nonzero coefficients in the LASSO regression model were also constructed in Fig. 3. This characteristic includes CTX, BMD, and ROM, and models using the aforementioned independent predictors were created and shown as nomograms. The nomogram demonstrated strong predictive performance, with a C-index of 0.925 (95% CI: 0.881–0.969) in the training cohort and 0.97 in the validation cohort, indicating excellent discriminatory power. The calibration curve showed close agreement between predicted and observed outcomes, suggesting high predictive accuracy. Decision curve analysis further confirmed the clinical utility of the nomogram, demonstrating that its use provides net benefit across a probability threshold range of approximately 12–82% for clinical decision-making in patients with SONK. This suggests our model offers superior clinical utility over both default treat-all and treat-none strategies within this range, supporting its practical value in guiding individualized clinical decision-making. The breach risk graph indicates a high degree of predictability. Figure 3 depicts the decision curve analysis for bone mass. The decision curves show that this non-compliant nomogram should be used to estimate the risk of bone mass in patients with SONK rather than this approach. When the patient and physician probability were more than 12 and less than 82%, respectively, based on the nonadherence risk nomogram, the net benefit was equivalent across this range, with numerous overlaps (Fig. 3).

## Discussion

Our study’s principal finding is that adjunctive denosumab therapy significantly improved functional and radiographic outcomes in patients with spontaneous osteonecrosis of the knee (SONK) undergoing UKA. At the 24-month follow-up, the denosumab group (Group B) demonstrated clinically meaningful and statistically superior results, including a 7-point improvement in HSS scores (transitioning from ‘poor’ to ‘good’ function), lower VAS pain scores, and greater range of motion (ROM) compared to controls. This was coupled with enhanced systemic bone health, evidenced by significantly higher BMD and T-scores, alongside a reduction in the bone resorption marker CTX, indicating denosumab’s dual benefit for both the operated joint and overall skeletal protection (37, 38).

When compared to complete knee replacement surgery, unilateral compartment joint replacement surgery provides benefits such as lower blood loss, greater adaptation to the body, improved range of motion, and enhanced feeling owing to cruciate ligament preservation (27, 39, 40, 41, 42, 43). Minimally invasive UKA producing a small amount of varus undercorrection in selected patients with medial tibiofemoral osteoarthritis or moderate avascular necrosis of the medial femoral condyle provides excellent clinical and functional results (44, 45). However, there are only a few indications for monocondylar arthroplasty. This procedure is generally recommended only for patients with femoral monocondylar involvement, no retropatellar osteoarthropathy, and stable lateral collateral ligaments, cruciate ligaments, knee deformity less than 22°, range of motion greater than 10°, no ligamentous laxity, and no obesity (46, 47, 48, 49). Late-stage SONK satisfies these characteristics, making it a suitable candidate for unicondylar knee replacement. Impaired fixation of monocondylar implants, on the other hand, leads to poor long-term results owing to inadequate bone reserve produced by osteonecrosis and risk factors, subsequent arthritic alterations, and osteonecrosis progression to other knee compartments (50, 51, 52).

Our findings suggest that denosumab, as an adjunct to UKA, offers a promising therapeutic strategy for SONK. This benefit may be attributed to its distinct mechanism of action compared to bisphosphonates, the more traditionally utilized antiresorptive agents. While bisphosphonates chemically bind to the bone mineral matrix and are internalized by osteoclasts, inducing apoptosis and thereby inhibiting bone resorption, their action is ultimately limited by their pharmacokinetic dependence on renal clearance and their permanent incorporation into the skeletal tissue (53). Denosumab, a fully human monoclonal antibody, exerts its effect by selectively inhibiting the receptor activator of nuclear factor kappa-B ligand (RANKL). This inhibition prevents the formation, function, and survival of osteoclasts at a more proximal regulatory point, resulting in a more rapid and profound suppression of bone turnover that is not dependent on renal function (54, 55, 56).

In terms of efficacy, clinical trials in osteoporosis have demonstrated that denosumab produces greater increases in BMD at all measured sites and a superior reduction in fracture risk at the vertebral and nonvertebral sites compared to most oral bisphosphonates (57). This potent antiresorptive effect is particularly relevant in the context of SONK, where rapid stabilization of the compromised subchondral bone is critical to prevent collapse and facilitate repair. The superior functional outcomes and higher BMD observed in our denosumab cohort align with this enhanced efficacy profile (58, 59).

Regarding safety, the two drug classes present different considerations. A well-documented risk associated with long-term bisphosphonate use is atypical femoral fracture (AFF), linked to oversuppression of bone turnover and accumulated microdamage (60). Denosumab treatment has not been associated with AFF. However, its reversible mechanism of action necessitates semiannual injections to maintain efficacy (61, 62, 63); upon discontinuation, a rapid rebound in bone resorption and a potential risk of multiple vertebral fractures have been observed, mandating careful patient management and follow-up (64, 65, 66, 67). Unlike bisphosphonates, denosumab is not contraindicated in patients with renal impairment, offering a significant advantage for elderly patients who often have compromised renal function (62, 68). In our study, the denosumab regimen was well-tolerated with no related adverse events reported, supporting its favorable safety profile for this application.

The death of osteocytes, which are progenitor cells of bone cells such as osteoblasts and osteoclasts, is referred to as osteonecrosis. It is often assumed that bone marrow stress or concealed subchondral bone fractures are to blame (69, 70, 71). Although cells die in necrotic lesions, the inorganic bone matrix initially stays relatively unharmed. However, ingrown blood arteries from neighboring live bone penetrate the necrotic bone, and osteoclasts are recruited to absorb the necrotic bone matrix (72). This starts the remodeling process, which includes both bone resorption and creation. Remodeled bone’s mechanical strength may be temporarily diminished owing to necrotic bone resorption or fatigue stress fractures in unrejuvenated necrotic bone (73). Necrosis in subchondral weight-bearing bone may lead to partial joint collapse and subsequent osteoarthritis (74).

Several animal studies back up the theory that limiting bone resorption following osteonecrosis retains the trabecular structure of the femoral head and reduces deformities (75). Bisphosphonates protect the trabecular structure and minimize femoral head deformities in rat and piglet models (76, 77). Bisphosphonates have also been demonstrated to slow the development of osteonecrosis in adults (78). Alendronate therapy was related to a considerable delay in femoral head collapse and a decreased requirement for complete hip replacement in a randomized clinical study (78).

The mechanism of action on osteoclasts differs between RANKL inhibitors (such as denosumab) and bisphosphonates (55). Bisphosphonates bind chemically to calcium hydroxyapatite in bone, limiting bone resorption by inhibiting osteoclast activity and survival. RANKL inhibitors, on the other hand, inhibit the interaction between RANKL and its receptor on osteoclasts, limiting their creation, function, and survival. When compared to bisphosphonates, denosumab has been found to have an equivalent or better ability to reduce bone turnover.

Denosumab decreases osteoclast and precursor development, activity, and survival by inhibiting the interaction of RANKL with its receptor RANK (79, 80). Bisphosphonates, on the other hand, chemically bind to calcium hydroxyapatite in bone and limit bone resorption by inhibiting the function and survival of osteoclasts (but not their formation) (81, 82). Denosumab has been proven to be as effective as or more effective than bisphosphonates in suppressing bone turnover (83, 84). Denosumab with UKA for SONK resulted in a decreased incidence of osteonecrosis and prosthesis loosening in our research compared to prior trials employing bisphosphonates plus UKA. Furthermore, our research discovered that denosumab treatments resulted in improved range of motion and lower pain levels.

Previous research has not included clinical trials of denosumab for the treatment of osteonecrosis. Guo *et al.* treated patients for 6 months, but we prolonged therapy until the central area exhibited an increase in radiological density, suggesting central osteoblast activity and possible mechanical strength recovery. The ideal treatment length is unknown (78). Jureus *et al.* proposed that an anticatabolic medication, bisphosphonate, that delays remodeling might be an effective therapy for osteonecrosis of the knee, although further randomized trials are needed (19).

Furthermore, the external validity (generalizability) of our findings is expressly limited to postmenopausal women with osteoporosis, as this was the exclusive population enrolled in our denosumab treatment group (85, 86, 87). Our study deliberately excluded male patients and those with secondary causes of osteoporosis to create a homogeneous cohort for initial evaluation. Consequently, the efficacy and safety of denosumab in these excluded populations, as well as in premenopausal women, remain unknown and warrant specific investigation in future studies. This limitation necessitates caution when considering the application of our results to a broader, more diverse patient population.

## Limitations

This study has several important limitations. First, as a prospective non-randomized study, the lack of randomization in treatment allocation and the absence of blinding for patients and outcome assessors may introduce potential selection bias and confounding. Second, the relatively small sample size is inherent to the low prevalence of spontaneous osteonecrosis of the knee (SONK), which may affect the statistical power of our findings. Third, the mean follow-up period of 2.1 years is insufficient to evaluate long-term arthroplasty outcomes, including implant survivorship, late-onset complications, and revision surgery rates. Fourth, while our predictive nomogram demonstrated excellent internal validity, it requires external validation in independent multicenter cohorts to confirm its generalizability and clinical utility. Finally, the study did not include a bisphosphonate treatment group for direct comparison, which would have provided valuable comparative efficacy data. Future large-scale, multicenter, randomized controlled trials with long-term follow-up are needed to validate these findings and establish robust evidence for this therapeutic strategy.

## Conclusion

The combination of denosumab and UKA demonstrates strong potential as a multimodal therapeutic strategy for SONK, significantly improving knee function and bone health while supporting its future integration into routine clinical practice. Further validation in larger, diverse cohorts is encouraged to confirm the generalizability of our predictive model and solidify denosumab’s role in standard care protocols.

## Declaration of interest

The authors declare no conflict of interest.

## Funding

This work did not receive any specific grant from any funding agency in the public, commercial, or not-for-profit sector.

## Author contribution statement

GPZ, KKP, YYZ, LL, and RJC conceptualized the study. YYZ, LL, and RJC were responsible for data curation, formal analysis, funding acquisition, investigation, methodology, and project administration. LL and RJC were responsible for resources, software, supervision, validation, visualization, writing the original draft, and writing review and editing.

## Data availability

The data used to support the findings of this study are included within the article.

## Ethics approval and consent to participate

This single-center controlled research was authorized by Longyan First Affiliated Hospital of Fujian Medical University’s ethical committee and institutional review board (IRB) (DJM0.2019.2.11). Informed consent to participate was obtained from all participants in the study. All human data must be in compliance with the Helsinki Declaration.

## Consent for publication

All authors agree to publication.
